# The associations between benevolent leadership, affective commitment, work engagement and helping behavior of nurses: a cross-sectional study

**DOI:** 10.1186/s12912-023-01581-6

**Published:** 2023-10-30

**Authors:** Xiaolin Shen, Tao Shen, Yanling Chen, Ying Wang, Xuan He, Xinyue Lv, Qiang Jin

**Affiliations:** 1 Department of Rheumatology and Immunology, West China Hospital, Sichuan University, Chengdu city, Sichuan Province People’s Republic of China; 2 School of Business Administration, Faculty of Business Administration, Southwestern University of Finance and Economics, Chengdu City, 610031 Sichuan Province People’s Republic of China; 3 Carey Business School, The Johns Hopkins University, 100 International Dr, Baltimore, MD 21202 USA; 4 International Business School, Southwestern University of Finance and Economics, Chengdu City, 610031 Sichuan Province People’s Republic of China; 5 Acupuncture School, Hospital affiliated to Chengdu University of Traditional Chinese Medicine, Chengdu City, Sichuan Province People’s Republic of China

**Keywords:** Benevolent leadership, Affective commitment, Work engagement, Helping behavior, Social exchange theory, Hospitals, Head nurses, Nurses

## Abstract

**Background:**

Benevolent leadership is common in organizations, including hospitals, and is known to have positive effects on employees. Yet, nursing literature lacks sufficient research on its relationships with nurses’ behavior.

**Methods:**

In March to April 2022, a cross-sectional study was carried out involving 320 nurses employed across various hospitals in Sichuan Province, China. Benevolent leadership, affective commitment, work engagement, and helping behavior were evaluated using the Benevolent Leadership Scale, Affective Commitment Scale, Work Engagement Scale, and Helping Behavior Questionnaire, respectively. The study employed structural equation model and the bootstrap method to investigate the proposed relationships.

**Results:**

The SEM analysis results indicated a positive association between benevolent leadership and several outcomes among nurses. Specifically, benevolent leadership was found to be positively associated with nurses’ affective commitment (*β =* 0.58, *p* < .001), work engagement (*β* = 0.02, *p* < .001), and helping behavior (*β* = 0.17, *p* = .001). Additionally, there was a significant indirect effect between benevolent leadership and nurses’ work engagement through affective commitment (*β =* 0.08, *p =* .007) as well as between benevolent leadership and helping behavior through affective commitment (*β =* 0.16, *p* < .001).

**Conclusions:**

This study’s findings emphasize the crucial role of benevolent leadership in fostering nurses’ positive attitudes and behaviors in the workplace. Hospital administrators could promote the benevolent leadership of head nurses to enhance nurses’ affective commitment, work engagement, and helping behaviors.

## Introduction

Leadership has been recognized as a critical factor that shapes the attitudes and behaviors of nurses [1–3], ultimately impacting the quality of patient service [4, 5]. The significance of leadership in nursing literature has been extensively studied, with scholars focusing on various forms of nurses’ behaviors such as innovative behaviors [6], service behaviors [7], voice behaviors [3], and proactive behaviors [8]. Furthermore, various leadership styles have also been investigated in nursing literature, including transformational leadership, authentic leadership, narcissistic leadership, and ethical leadership. These styles have consistently shown positive associations with nurses’ outcomes [9, 10]. For instance, [11]’s research established a positive correlation between transformational leadership and nurses’ job satisfaction. Additionally, [12]’s study provided evidence that ethical leadership serves as a strong predictor of nurses’ engagement in organizational citizenship behaviors. However, despite the growing body of research on the impact of different types of leadership on nurses’ behaviors, benevolent leadership, which refers to the personalized attention and care that leaders provide to their subordinates, has received limited attention in nursing literature [13, 14].

In the organizational management literature, benevolent leadership has been shown to have positive effects on subordinates, including improving psychological well-being [13], creativity [14], taking charge [15], and organizational citizenship behaviors [16]. Despite its prevalent existence in contemporary organizations, including hospitals [17, 18], the impact of benevolent leadership on nurses’ working behaviors has been largely neglected in the nursing literature. Given that benevolent leadership has been demonstrated to play a pivotal role in influencing employees’ outcomes in previous research, there exists a compelling need to delve into how benevolent leadership specifically affects nurses’ outcomes within the nursing profession. Further exploration of the impact of benevolent leadership on nurses’ working outcomes can contribute valuable insights to the nursing literature and potentially inform leadership practices in healthcare settings.

In this study, we aim to address the current gap in the literature by examining the influence of benevolent leadership on nurses’ positive behaviors, with a focus on work engagement and helping behavior as potential outcomes of the benevolent leadership of head nurses. Work engagement and helping behavior are critical to improving nurses’ and overall work unit performance [19, 20]. Work engagement, which is defined as a positive state of mind in the workplace and comprises absorption, vigor, and dedication [21], has consistently been found to be a significant predictor of employee performance [22, 23]. For instance, a study found that work engagement is positively associated with innovative behaviors among nurses [14]. Moreover, a meta-analysis indicated that work engagement is positively related to patient quality of care [24]. Helping behavior, on the other hand, refers to voluntary actions that assist colleagues in avoiding or resolving problems at work [25]. These behaviors may take the form of altruism, care, and cooperation [25, 26] and have long been identified as a significant predictor in promoting group cohesion and interpersonal harmony, ultimately enhancing nursing service quality. Helping behavior reflects a proactive approach where nurses voluntarily assist their colleagues, ultimately contributing to a positive work atmosphere and patient care quality. Despite the significance of work engagement and helping behavior in the nursing profession, research on the relationship between benevolent leadership and these two behaviors is limited, thus providing a strong motivation to investigate the relationships between them. Drawing on social exchange theory, we hypothesize that head nurses’ benevolent leadership is positively associated with both within-person behavior (i.e., work engagement) and between-person behavior (i.e., helping behavior) among nurses.

Social exchange theory suggests that subordinates tend to reciprocate their leaders by exhibiting positive working attitudes and engaging in positive work behaviors when they perceive that their leaders treat them well [27, 28]. Accordingly, we propose that nurses are more likely to perform positive behaviors when they perceive that their head nurses lead them with benevolent leadership, which includes demonstrating care for them, mentoring them, and solving problems for them. Specifically, we categorize the potential positive behaviors brought about by benevolent leadership into within-person behavior (work engagement) and between-person behavior (helping behavior), and thus propose that head nurses’ benevolent leadership is significantly and positively related to nurses’ work engagement and helping behavior.

To gain a deeper understanding of the intricate relationship between benevolent leadership and nurses’ positive behaviors, it is imperative to explore the underlying mechanisms driving this connection. Drawing on the foundational framework of social exchange theory and related literature, we propose that nurses’ affective commitment serves as a pivotal mediating factor in the association between benevolent leadership and their work engagement and helping behavior. Affective commitment, as delineated in the literature [29], encompasses the emotional facets of identification, attachment, and engagement that individuals harbor toward their organization. We chose affective commitment because it reflects strong emotional bonds with an organization or leader. It aligns closely with benevolent leadership’s care and support, offering a direct route for influencing nurses’ behavior. Furthermore, affective commitment boosts productivity, reduces absenteeism, and shapes organizational citizenship behavior [30, 31]. Its role as a mediator between leadership styles and outcomes is empirically supported, contributing to our understanding of how benevolent leadership affects nursing behavior. Nurses who exhibit a heightened level of affective commitment are not only more likely to approach their work with fervor and dedication but also less inclined to consider leaving their current healthcare establishments [32].

In accordance with the tenets of social exchange theory, benevolent leaders, through their compassionate and caring leadership style, are apt to foster a deep-seated psychological attachment and commitment among their subordinates toward the organization. Consequently, we hypothesize that benevolent leadership’s initial impact will be on nurses’ positive work attitudes, primarily their affective commitment. This, in turn, is expected to trigger a cascade of positive work behaviors, including heightened work engagement and an increased propensity for engaging in helping behavior. Affective commitment, by its very nature, can act as a catalyst for employees’ unwavering dedication to making substantial contributions to their respective organizations [33]. Previous research has provided some empirical evidence that affective commitment has positive relationship with work engagement and helping behavior [34, 35].

Based on the social exchange theory and related literature, we propose the following hypotheses: H1: Benevolent leadership is positively related to nurses’ affective commitment; H2a: Benevolent leadership is positively related to nurses’ work engagement; H2b: Benevolent leadership is positively related to nurses’ helping behavior; H3a: Nurses’ affective commitment mediates the relationship between benevolent leadership and nurses’ work engagement; H3b: Nurses’ affective commitment mediates the relationship between benevolent leadership and nurses’ helping behavior.

The aim of this paper is to examine the influence of head nurses’ benevolent leadership on nurses’ work engagement and helping behavior, mediated by nurses’ affective commitment.

## Methods

### Study design and sample

A questionnaire-based cross-sectional study was conducted to investigate the relationship between benevolent leadership and nurses’ work engagement and helping behavior, as well as the mediating role of affective commitment in these relationships. The data were collected between March and April 2022.

The sample size calculation was performed using G*Power 3.1. Specifically, together with eight control variables, there were a total of ten predictors. We opted for linear multiple regression with a fixed model and R² deviation from zero as our statistical test, employing F-tests. Subsequently, we selected an effect size of 0.15, an α error probability of 0.05, and a power (1-β error probability) of 0.95. Theoretically, a minimum sample size of 172 was required. We utilized convenience sampling and recruited 343 nurses who were currently working in tertiary and secondary hospitals in Sichuan Province, China. All participants had at least one year of experience as a nurse. The inclusion criteria were as follows: (a) registered nurses; (b) understanding the purpose of the study; (c) able and willing to participate; and (d) presently working in their current ward. The survey link was disseminated among the participants via the social media platform, WeChat. In order to incentivize participation, a monetary compensation of 10 RMB was offered to those who completed the questionnaire to a satisfactory standard. Out of the 343 questionnaires distributed, 325 (94.8%) were completed. After excluding five questionnaires due to incomplete data and illogical answers, data from 320 (93.3%) participants were included in the final data set, exceeding the minimum sample size of 172.

### Measures

Demographic data, including gender, age, education level, marital status, income, hospital level, years of working experience in the hospital, and positional rank, was collected. The four main study variables, benevolent leadership, affective commitment, work engagement and helping behavior, were measured by the instruments described below. Since the original measurements were English, we performed translation and back-translation procedures between English and Chinese to ensure content validity [34]. Specifically, the initial translation of the scales from English to Chinese was conducted by two highly skilled individuals, both of whom were professors with expertise in English and nursing terminology. Moreover, back translation was carried out with the support of two additional experts possessing similar language proficiency and familiarity. To evaluate face validity, a panel consisting of three registered nurses and two Ph.Ds of management were invited to meticulously review and provide feedback on the translated materials. Additionally, to ensure the content validity of the scales, three experts in the fields of research and instrument design were approached to contribute their valuable insights and recommendations about the scales.

#### Benevolent leadership

In this study, benevolent leadership was measured by eleven items from [35]’s Benevolent Leadership Scale. Sample items include “My supervisor is like a family member when he/she gets along with us”, “My supervisor devotes all his/her energy to taking care of me”, and “Beyond work relations, my supervisor expresses concern about my daily life”. The participants selected the degree of agreement from a five-point Likert scale which ranges from 1 which represents “strongly disagree” to 5 which stands for “strongly agree”. Its reliability Cronbach’s alpha coefficient in this study is 0.978.

#### Affective commitment

Affective commitment was assessed by three items Affective Commitment Scale from [36]. Items include “I am glad to spend my career in this organization”, “I am glad to tell other people about my workplace”, and “I felt like I am a part of this big family”. The scores on the scale also range from 1 = strongly disagree to 5 = strongly agree. Its Cronbach’s alpha in this study is 0.953.

#### Work engagement

Work engagement was measured in this study using six items Work Engagement Scale by [37]. Sample items include “I work with intensity on my job”, “I exert my full effort on my job”, and “I devote a lot of energy to my job”. The scale scores range from 1 = strongly disagree to 5 = strongly agree. Its Cronbach’s alpha in this study is 0.967.

#### Helping behavior

Helping behavior was evaluated using four items Helping Behavior Scale from [38]. Sample items include “I helped other group members with their work responsibilities”, “I assisted other group members in their work for the benefit of the group”, and “I got involved to benefit the group”. The scores on the scale also range from 1 = strongly disagree to 5 = strongly agree. Its Cronbach’s alpha in this study is 0.962.

### Ethical considerations

The institutional review board of the first author’s affiliated hospital in Sichuan Province, China approved this study (IRB No. 2,022,572). All methods were performed in accordance with the Declaration of Helsinki and other relevant guidelines and regulations. The informed consent to participate in the study were obtained from participants. The results of this study has no effect on the participants. Participants were told that they could choose to voluntarily participate in this study and that they could withdraw from the survey at any time. We informed the participants that the collected data would only be used for research purposes and that their information was anonymous and would be kept confidential.

### Data Analysis

We adopted AMOS 26.0 as the analytical tool and ran structural equation model (SEM) to test the hypothesized relationships in this paper. The demographic characteristics of the participants were reported using descriptive statistics. Pearson’s correlation coefficient was adopted to analyze the correlations among key study variables. The normality of the distributions was assessed using the Kolmogorov-Smirnov test. This test was conducted at a 95% confidence level, meaning that if the significance level value is less than 0.05, the distribution is not considered normal. In this study, since the *p*-values were greater than 0.05, it indicates that the variables followed a normal distribution. All statistical results were set at a significance level of *p* < .05.

## Results

### Descriptive characteristics

Table 1 provides an overview of the demographic characteristics of the 320 participants. The majority of the participants were female (303, 94.7%), while only 17 (5.3%) were male. In terms of age, 45.6% of the participants were younger than 30 years old, 47.5% were aged between 31 and 40 years, and 6.9% were 41 years or older. Regarding marital status, 100 (28.8%) were single, while 220 (68.7%) were married. Most of the participants (81.9%) had attained a bachelor’s degree or higher, while 82.8% reported an annual income between ¥50,000 and ¥200,000. In terms of work experience, 39.7% of the participants had five years or less of experience in hospital, 36.3% had between five and ten years of experience, and 24% had more than ten years of experience in hospital. In terms of positional rank, 97.5% were general nurses, while the rest were charge nurses.

**Table 1:** Participant demographic variables (N = 320)

Variables	n	%
**Gender**		
Male	17	5.3
Female	303	94.7
**Age (years)**		
≤ 30	146	45.6
31–40	152	47.5
41–50	17	5.3
≥ 51	5	1.6
**Marital status**		
Married	220	68.7
Single	92	28.8
Divorced	8	2.5
**Educational level**		
Low (college education and below)	58	18.1
High (bachelor’s degree and above)	262	81.9
**Annual income**		
Low (< ¥50,000)	44	13.8
Middle (¥50,000–¥200,000)	265	82.8
High (> ¥200,000)	11	3.4
**Hospital level**		
Secondary	63	19.7
Tertiary	257	80.3
**Years in practice**		
Low (< 5)	127	39.7
Middle (5–10)	116	36.3
High (> 10)	77	24
**Nursing position**		
General nurse	312	97.5
Charge nurse	8	2.5

### Correlations among benevolent leadership, affective commitment, work engagement, and helping behavior

Table 2 summarizes the correlations among the variables. Benevolent leadership was found to be significantly and positively correlated with affective commitment (*r* = .65, *p* < .01), work engagement (*r* = .24, *p* < .01), and helping behavior (*r* = .49, *p* < .01). This indicates that benevolent leadership may contribute to higher levels of affective commitment, work engagement, and helping behavior among nurses.

**Table 2:** Means, standard deviations (SD), and correlation coefficients of key study variables (N = 320)

Variables	Mean	SD	Benevolent leadership	Affective commitment	Work engagement	Helping behavior
Benevolent leadership	3.82	0.86	1.00			
Affective commitment	4.08	0.74	0.65**	1.00		
Work engagement	4.78	0.49	0.24**	0.29**	1.00	
Helping behavior	4.44	0.60	0.49**	0.49**	0.55**	1.00

**p < .01

Furthermore, a positive and significant correlation was observed between affective commitment and both work engagement (*r* = .29, *p* < .01) and helping behavior (*r* = .49, *p* < .01). This suggests that nurses who are more emotionally attached to their organization are more likely to exhibit higher levels of work engagement and helping behavior.

Lastly, work engagement was found to be significantly and positively correlated with helping behavior (*r* = .55, *p* < .01). This finding implies that employees who are more invested in their work and experience higher levels of vigor, dedication, and absorption are more likely to engage in helping behavior.

### Hypothesis testing

#### Testing the relationship between benevolent leadership and nurses’ affective commitment (H1)

We employed SEM to test the hypotheses. Several criteria were employed to assess the adequacy of model fit, including the Ratio of Chi-Square to Degrees of Freedom (χ²/df), Comparative Fit Index (CFI), Tucker-Lewis Index (TLI), and the Root Mean Square Error of Approximation (RMSEA). To qualify as a well-fitting model, it was imperative for the χ²/df value to remain below 3, while both CFI and TLI had to attain a minimum threshold of 0.90 or higher. Additionally, a favorable model fit was indicated by an RMSEA value below 0.08 [39]. The assessment of the measurement model involved a thorough examination through confirmatory factor analysis (CFA), aiming to gauge the adequacy of how the observed items represented latent variables and the extent to which the fundamental constructs were distinguishable from one another. The results of the CFA are showed in Table 3. Remarkably, the measurement model displayed good data fit, as indicated by the following fit indices: χ2(239) = 648.22 (*p* < .001), CFI = 0.96, TLI = 0.96, RMSEA = 0.07. The assurance of convergent validity for the constructs was affirmed by assessing the item loadings and their corresponding significance. The data analysis showed that the cross-loadings of items onto their respective constructs, spanning a range from 0.733 to 0.982, consistently exceeded the recommended minimum threshold of 0.5 and exhibited statistical significance. This observation underscores the presence of convergent validity for the constructs, aligning with the principles outlined by [39].

**Table 3:** The results of CFA

*Constructs and items*	*Cross-loadings*
*Benevolent leadership (BL)*	
*BL1*	0.888***
*BL2*	0.904***
*BL3*	0.911***
*BL4*	0.927***
*BL5*	0.870***
*BL6*	0.887***
*BL7*	0.859***
*BL8*	0.807***
*BL9*	0.946***
*BL10*	0.913***
*BL11*	0.918***
*Affective commitment (AC)*	
*AC1*	0.924***
*AC2*	0.928***
*AC3*	0.949***
*Work engagement (WE)*	
*WE1*	0.733***
*WE2*	0.963***
*WE3*	0.894***
*WE4*	0.982***
*WE5*	0.973***
*WE6*	0.953***
*Helping behavior (HB)*	
*HB1*	0.899***
*HB2*	0.941***
*HB3*	0.940***
*HB4*	0.901***

***p < .001; χ2(239) = 648.22 (*p* < .001), CFI = 0.96, TLI = 0.96, RMSEA = 0.07.

**Fig. 1:**
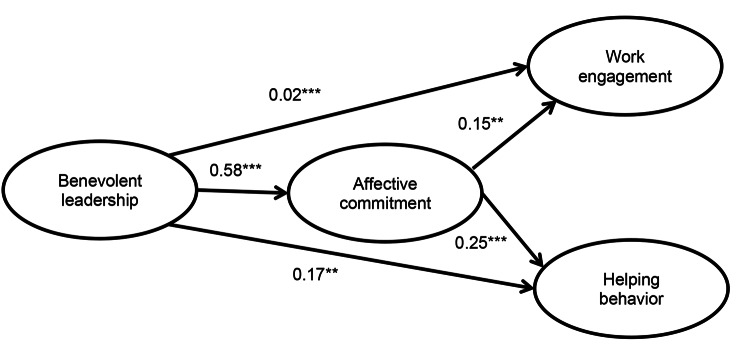
Results of structural equation modeling. **p < .05; ***p < .001; χ2(239) = 677.62 (*p* < .001), CFI = 0.96, TLI = 0.95, RMSEA = 0.07

The hypothesized model underwent examination through the utilization of maximum likelihood analysis in SEM. The findings are presented in Table 4; Fig. 1. Adequate model fit was indicated by the following fit indices: χ2(239) = 677.62 (*p* < .001), CFI = 0.96, TLI = 0.95, and RMSEA = 0.07. Hypothesis 1 posited benevolent leadership has a positive relationship with nurses’ affective commitment. The results of SEM analysis revealed that benevolent leadership was positively and significantly related to nurses’ affective commitment (*β* = 0.58, *p* < .001). Thus, the results supported H1. This finding emphasizes the importance of benevolent leadership in healthcare. Head nurses, by adopting a benevolent leadership style that demonstrates care and empathy, can enhance nurses’ commitment to the organization. This emotional attachment and loyalty can lead to improved job satisfaction, reduced turnover, and better overall performance among nurses. Thus, healthcare organizations should encourage and develop benevolent leadership among their head nurses to create a more supportive work environment and benefit both nurses and the organization.

**Table 4:** Standardized direct and indirect effects of hypothetical model (N = 320)

Path	β	*p*	95% CI [Lower, Upper]
Direct effect			
Benevolent leadership -->Affective commitment	0.58	< 0.001	[0.474, 0.688]
Benevolent leadership -->Work engagement	0.02	< 0.001	[0.058, 0.117]
Benevolent leadership -->Helping behavior	0.17	0.001	[0.066, 0.268]
Indirect effect			
Benevolent leadership -->Affective commitment–>Work engagement	0.08	0.007	[0.022, 0.167]
Benevolent leadership -->Affective commitment–>Helping behavior	0.16	< 0.001	[0.076, 0.250]

CI = confidence interval

#### Testing the relationship between benevolent leadership and nurses’ work engagement (H2a)

Moreover, the results of SEM analysis also demonstrated that benevolent leadership was positively associated with nurses’ work engagement (*β* = 0.02, *p* < .001), lending support to H2a, which proposed that benevolent leadership is positively associated with nurses’ work engagement. This finding suggests that head nurses’ benevolent leadership style may contribute to enhancing nurses’ levels of vigor, dedication, and absorption in their work.

#### Testing the relationship between benevolent leadership and nurses’ helping behavior (H2b)

Furthermore, the results revealed that benevolent leadership had a positive association with nurses’ helping behavior (*β* = 0.17, *p =* .001), which corroborates H2b, positing that benevolent leadership is positively related to nurses’ helping behavior. This implies that head nurses’ benevolent leadership style may encourage nurses to engage in activities aimed at supporting their colleagues. The findings in H2a and H2b emphasize the importance of benevolent leadership in healthcare organizations. Cultivating a leadership style marked by care, empathy, and support positively influences nurses’ work engagement and their willingness to help others. To implement these findings, organizations should training leaders in benevolent leadership skills. This approach can enhance nurse performance, foster collaboration, and ultimately improve patient care.

#### Testing for the mediation of affective commitment (H3a and H3b)

The mediating roles of affective commitment were examined by 5000 bootstrap analyses with 95% confidence intervals. As shown in Table 4, the indirect effect of benevolent leadership → affective commitment → work engagement was 0.08 (*p* = .007), which suggested that affective commitment mediates the relationship between benevolent leadership and work engagement. Thus H3a was supported. Likewise, the indirect effect of benevolent leadership → affective commitment → helping behavior was 0.16 (*p <* .001), indicating that affective commitment mediates the relationship between benevolent leadership and helping behavior, supporting H3b. The findings in H3a and H3b confirmed that affective commitment plays a pivotal role as a mediator between benevolent leadership and both work engagement and helping behavior among nurses. Encouraging leaders to demonstrate benevolence and support can enhance nurses’ emotional attachment which in turn affects job engagement and teamwork. Healthcare organizations should provide leadership training that fosters these qualities, ultimately benefiting patient care and overall organizational effectiveness.

To test the robustness of our model, we ran sub-group SEM based on marital status to explore whether the impacts of benevolent leadership on work engagement and helping behavior would be different. Both sub-group met the adequate model fit by the following fit indices for married nurses: χ2(239) = 533.16 (*p* < .001), CFI = 0.96, TLI = 0.96, and RMSEA = 0.07, and for unmarried nurses: χ2(239) = 550.89 (*p* < .001), CFI = 0.92, TLI = 0.91, and RMSEA = 0.07. In the married nurses group, the association between benevolent leadership and work engagement (*β* = 0.013, *p* < .001) closely paralleled that of unmarried nurses (*β* = 0.035, *p* < .001). However, the connection between benevolent leadership and helping behavior (*β* = 0.227, *p* < .001) was slightly stronger for married nurses compared to unmarried nurses (*β* = 0.032, *p* < .001). This difference may be attributed to married nurses having a higher sense of responsibility and altruistic spirit than their unmarried counterparts. In summary, the impact of leadership on both work engagement and helping behavior remained consistent across different subgroups based on marital status. This reinforces the validity of our results.

## Discussion

The present study investigated the relationship between benevolent leadership and nurses’ work engagement and helping behavior. The findings indicate that a benevolent leadership style has a positive influence on both nurses’ work engagement and helping behavior. Moreover, nurses’ affective commitment was found to mediate the relationship between benevolent leadership and both work engagement and helping behavior. These findings align with social exchange theory, which posits that employees are likely to reciprocate positive treatment from leaders by exhibiting positive behaviors [27].

Specifically, our study revealed a significant and direct relationship between benevolent leadership and affective commitment. To our knowledge, this is among the first studies that established the link between benevolent leadership and nursing staff’s outcomes. This finding supports the argument that employees’ affective commitment is largely influenced by the leadership styles of their supervisors [40]. Our results suggest that head nurses’ benevolent leadership can increase nurses’ commitment levels toward the hospital where they work. This finding is consistent with previous research that also reported a positive relationship between benevolent leadership and affective commitment [41, 42]. However, what this paper differs from the previous studies is that we found a positive relationship between benevolent leadership and affective commitment in the context of nursing rather than the normal organizations. Collectively, these findings highlight the significant role of benevolent leadership in enhancing organizational members’ psychological attachment to their workplace.

Additionally, our study revealed that benevolent leadership is positively associated with nurses’ work engagement and helping behavior, providing further support for the crucial role of benevolent leadership in promoting positive employee behaviors. The extant literature reports both positive and negative outcomes of benevolent leadership. For example, prior studies have shown that benevolent leadership enhances employees’ creativity, well-being, and taking charge [13, 15, 35], but it can also trigger employees’ unethical behaviors [43]. In contrast to the findings of negative relations between benevolent leadership and employees’ outcomes [43], this study indicated a positive one between benevolent leadership and nurses’ behaviors, thus contributing to the benevolent leadership literature by providing evidence that supports its positive impact on nursing practitioners.

The positive correlation between benevolent leadership and work engagement has been consistently observed in the literature, as evidenced by [44] and [15], who both reported improvements in employees’ work engagement due to benevolent leadership. The antecedents of work engagement has been widely studied in nursing literature. Scholars have found factors such as job satisfaction, practice environment, and leadership, to play as predictors of work engagement of nurses [30, 45, 46]. This study adds new knowledge to this line of research by identifying a new predictor which is benevolent leadership, Social exchange theory suggests that the supportive nature of leaders can influence employee behavior, as employees tend to reciprocate supportive leaders by engaging in positive behaviors. As a response to the benevolent leadership of head nurses, nurses may work more passionately and exhibit greater engagement in their job position, expecting to reciprocate their head nurses and contribute to the welfare of their work units.

In the nursing literature, the positive relationship between benevolent leadership and helping behavior found in this study appears to be the first empirical evidence of its kind. While previous studies have examined the relationship between benevolent leadership and organizational citizenship behavior [16], the specific relationship between benevolent leadership and helping behavior has received limited attention. This study bridges this gap by establishing a positive correlation between benevolent leadership and the helping behavior of nurses.

This study contributes to the nursing literature in several significant ways. First, our findings provide pioneering empirical evidence of the positive association between benevolent leadership and nurses’ work engagement and helping behavior, thereby highlighting the role of benevolent leadership in enhancing the positive behaviors of nurses. Second, our study identifies the mediating role of affective commitment in transforming benevolent leadership into nurses’ positive behaviors, which contributes to the extant literature on benevolent leadership.

The practical implications of our findings are particularly relevant for nursing practice. Hospital administrators can consider cultivating head nurses’ benevolent leadership style to enhance nurses’ positive working behaviors, such as work engagement and helping behavior. To this end, hospital administrators can provide training to head nurses, encouraging them to provide opportunities for subordinates to correct mistakes, provide more mentoring, avoid public humiliation, and solve subordinates’ working problems. Hospitals can develop and implement specialized leadership training programs for head nurses that specifically focus on cultivating benevolent leadership qualities. These programs can include modules on empathy, communication, and conflict resolution to enhance their leadership skills. Furthermore, hospitals can integrate benevolent leadership qualities as a component of the evaluation process for leadership promotions by including criteria related to empathy, supportiveness, and team building when assessing candidates for leadership positions.

Additionally, our study highlights the necessity of cultivating nurses’ affective commitment, which can be achieved through fostering a positive work culture, providing constructive feedback, communicating clear goals and expectations to employees, and encouraging open communication. Specifically, head nurses should recognize and appreciate the dedication and efforts of nursing staff. Hospitals can implement recognition programs that celebrate nurses’ contributions and achievements since personalized recognition can deepen affective commitment. Additionally, hospitals can regularly conduct employee engagement surveys to assess the level of affective commitment among nursing staff and use the feedback to identify areas where affective commitment can be strengthened and tailor leadership approaches accordingly. Furthermore, head nurses can facilitate mentorship programs where experienced nurses can mentor newer staff. Affective commitment often grows when nurses feel supported and have experienced colleagues to turn to for guidance.

### Limitations

While our study contributes novel insights into the positive relationship between benevolent leadership and nurses’ positive behaviors, several limitations should be acknowledged. Firstly, our sample of nurses was drawn from convenience sampling within a single province in China, which may limit the generalizability of our results to other settings. Cultural and contextual factors, such as regional variations in healthcare practices, communication norms, and patient-nurse dynamics, specific to this province, could potentially influence our findings. Additionally, variations in healthcare infrastructure, government policies, and socioeconomic conditions across different provinces in China or in the world may also introduce confounding factors that affect the generalizability of our conclusions beyond the boundaries of the sampled province. Therefore, while our study provides valuable insights, it is essential to exercise caution when extrapolating these findings to a more diverse and global healthcare context.

Secondly, we recognize the possibility of self-report bias and its potential to introduce inaccuracies into our data due to the reliance on self-reported measures. However, we took proactive steps to mitigate this bias by implementing an anonymous questionnaire design. This approach aimed to create a confidential and non-judgmental environment, encouraging participants to provide honest and unfiltered responses. By ensuring anonymity, we sought to minimize the likelihood of response bias and enhance the reliability of the data collected.

Thirdly, while our cross-sectional study design precludes the establishment of causal relationships among the key variables, it is essential to acknowledge its inherent limitations. The nature of our study design primarily allows for the examination of associations and correlations rather than causation. Therefore, caution should be exercised when interpreting the findings in terms of cause-and-effect relationships. Additionally, it is important to recognize the potential presence of confounding factors or reverse causation that may influence the observed relationships. Factors not considered in this study, such as individual differences in personality traits, work-related stressors, or external environmental factors, could impact both the leadership styles employed by head nurses and the outcomes measured among nursing staff. Future research endeavors should aim to employ longitudinal or experimental designs that can provide more robust evidence regarding the causal relationships between benevolent leadership, affective commitment, work engagement, and helping behavior. Furthermore, conducting in-depth analyses to identify and control for potential confounding variables can enhance the validity and generalizability of the findings.

Finally, while our study identifies affective commitment as a mediator between benevolent leadership and nurses’ positive behaviors, we should acknowledge the potential existence of other mediators such as trust, leader-member exchange, or job satisfaction of nurses. These alternative mechanisms should be explored in future research to gain a more comprehensive understanding of this relationship. Investigations into these potential alternative mediators will provide deeper insights for the benevolent leadership research in nursing. Overall, these limitations suggest opportunities for future research to extend our understanding of the relationship between benevolent leadership and nurses’ positive behaviors. By addressing these limitations, future studies could enhance the generalizability, accuracy, and causality of the relationship between benevolent leadership and nurses’ positive behaviors.

## Conclusions

To summarize the key takeaways and emphasize the significance of our study, it is essential to highlight that benevolent leadership, while prevalent in organizations, has been underexplored in the nursing literature regarding its impact on nurses’ behaviors. Our research addresses this critical gap by examining the influence of benevolent leadership on nurses’ work engagement and helping behavior, with affective commitment acting as a mediating mechanism. Our findings robustly demonstrate a positive association between head nurses’ benevolent leadership and both within-person behavior, represented by work engagement, and between-person behavior, reflected in helping behavior among nurses. Furthermore, our study underscores the pivotal role of affective commitment as the mechanism linking benevolent leadership to these positive behaviors. This paper makes valuable contributions to the field of leadership in nursing literature. Practically, this study provides a valuable avenue for hospital administrators, who can promote benevolent leadership among head nurses to bolster nurses’ affective commitment, work engagement, and helping behavior, ultimately contributing to the delivery of high-quality patient care. Moving forward, future research in the field of benevolent leadership and nursing behaviors should explore additional factors and contexts to provide a more comprehensive understanding of this leadership style’s effects on nursing outcomes. Future research can consider exploring the alternative mechanisms between benevolent leadership and nurses’ outcomes. Furthermore, future research can also examine other outcomes other than the two investigated in this study with a longitudinal study design.

## Data Availability

The datasets analyzed during the current study are available from the corresponding author on reasonable request.
